# Kangaroo Mother Care for Low-Birth-Weight Babies in Low and Middle-Income Countries: A Narrative Review

**DOI:** 10.7759/cureus.38355

**Published:** 2023-04-30

**Authors:** Vaishnavi D Dhage, Asmita Rannaware, Sonali G Choudhari

**Affiliations:** 1 School of Epidemiology and Public Health, Jawaharlal Nehru Medical College, Datta Meghe Institute of Higher Education and Research, Wardha, IND

**Keywords:** premature birth, newborn death, neonatal care, middle-income countries (mics), low-income countries (lics), infant mortality, low birth weight (lbw), kangaroo mother care (kmc)

## Abstract

In low and middle-income countries (LMICs), the infant mortality rate is much higher than the high-income countries (HICs). The higher infant mortality is due to low birth weight (LBW) a combination of intra-uterine growth retardation (IUGR) and prematurity, which are risk factors for acquiring infectious diseases amongst newborns. Kangaroo mother care (KMC) is a neonatal procedure that is carried out in newborn infants, especially in preterm babies and LBW babies. It is skin-to-skin contact between a mother’s bare chest and a stable infant. KMC is an important intervention in reducing infant mortality rates in LMICs. A comprehensive literature and data search was done using key databases like PubMed and Google Scholar. A total of 42 articles out of 1,168 articles were selected for review after screening and elimination of the repeated articles. Through this review we have tried to analyse the benefits of KMC in newborns, the need for the participation of fathers and family members, and the need for implementation of this practice at a broader level by policy formulation in LMICs. We have also discussed the need for KMC for the prevention of infant mortality in LBW newborns in LMICs.

## Introduction and background

Low-birth-weight (LBW) infants, those born prematurely or undersized for gestational age, make up roughly 15% of newborns yet are responsible for 70% of all neonatal mortality. The Sustainable Development Goals (SDGs) aim to lower newborn mortality to less than 12/1000 live births in each country by 2030. This depends on reducing fatalities in LBW infants, especially in low and middle-income countries (LMICs) of Sub-Saharan Africa and Asia [1,2]. Reducing newborn deaths is crucial in attaining the Millennium Development Goal (MDG) 4 to reduce infant mortality [3].

In LMICs like South Africa and Vietnam, kangaroo mother care (KMC) begins soon after birth in LBW infants leading to an earlier stabilization than standard treatment [4]. KMC adoption has been moderate in some LMICs [5]. The ratio of LBW infant mortality is greater in LMICs as compared to high-income countries (HICs). KMC is skin-to-skin contact between a parent, especially a mother’s bare chest, and a medically stable LBW infant weighing less than 2500 g [6]. Babies are considered to be very low birth weight (VLBW) if they weigh less than 1,500 g, and extremely low birth weight (ELBW) if they weigh less than 1,000 g [7]. KMC encourages breastfeeding, effective heat regulation, prevention of infections, and bonding between mother and child, all of which are beneficial to their health and well-being. It is an easy and efficient way to upgrade the health and growth of premature LBW infants and full-term infants [8]. In 1979, Dr. Hector Martinez and Dr. Edgar Rey, both pediatricians working at Bogota Hospital in Colombia, first introduced LBW care by adopting KMC [9]. In the year 1983, the United Nations Children's Fund (UNICEF) launched this technique in other nations including LMICs [9].

KMC largely came up with tactical solutions to make up for the lack of facilities for treating LBW babies [10]. The KMC method, which promotes breastfeeding and bonding by placing premature and LBW babies against the mother's chest, is used all around the world [11]. No special equipment is required for KMC. The KMC technique aids in nurturing preterm/LBW babies and increases the baby's level of protection [12]. Premature birth and restricted intrauterine growth are the causes of LBW. 

The infant is placed on the mother's bare chest, which is typically swaddled in a warm quilt, in the KMC method [13]. The adoption of KMC as a substitute for traditional practices in LBW infants can be useful in reducing the risk of infant mortality [14]. In rural India, the most frequently conducted traditional practices include giving sugar water to the baby instead of colostrum as a first food, applying black dots (kohl) to newborns for their protection from evil power, and many more. KMC can be done both during the day and night [15]. The main goal is to investigate the idea that encouraging KMC in LMICs enhances LBW infants’ ability to effectively nurse during the neonatal period or 28 days following the delivery of the baby. Growth (counted as weight gain), decreased risk of potentially serious bacterial illness, increased exclusive breastfeeding, and continuing breastfeeding practices are the secondary goals [16].

Low birth weight, malnutrition, childhood infections, impairment, and deformity are typical children's health issues. Discussing the issue's magnitude along with the finest ways in nurturing LBW/preterm newborns should be a part of continuous neighborhood activities [17]. The mother maintains the infant’s life by performing constant KMC [18].

In several LMICs, a small portion of LBW babies can obtain healthcare supervision in infirmaries. Some deliveries occur at home and infants delivered in hospitals are frequently discharged before the suggested dates. Consequently, it is crucial to evaluate KMC completely and effectively [19]. The first 24 hours after birth are stressful for LBW newborns. The WHO recommends the KMC should be provided to infants weighing 2,500g or less [20]. Twenty million LBW neonates are born worldwide [6]. As per the WHO, preterm birth along with low birth weight are the major reasons for neonatal mortality [21].

## Review

Methodology 

**Figure 1:**
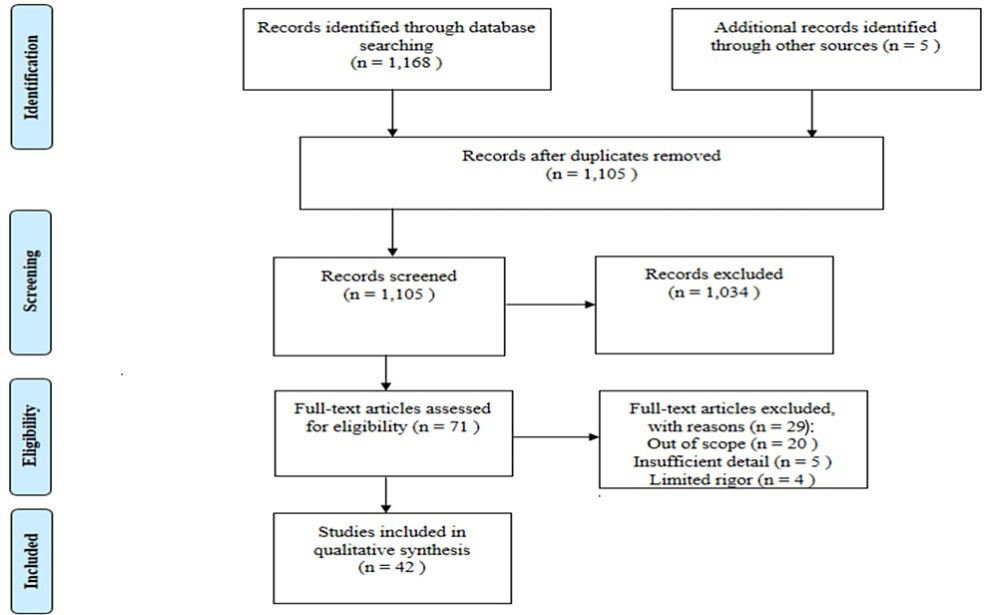
Flowchart of selection of studies/articles for the review

This review discusses KMC in LMICs. We searched PubMed/MEDLINE (Medical Literature Analysis and Retrieval System Online, or MEDLARS Online), Google Scholar, and WHO Data Collections in November 2022 by using medical subject headings (MeSH) terms like Kangaroo Mother Care and Lowbirth Weights. We additionally searched for key references from references of the relevant studies. The language of the study is English, and studies in other languages were excluded. The various studies published from 2000 to 2022 were included in the review. Additional filters such as free full text and full text were applied during the search process for this review. For more detail, we used keywords such as "Preterm births," "Infant mortality," "Child Health," "National Health Programs for Infants," "Low-Income Countries," and "Middle-Income Countries." The inclusion and exclusion criteria for selection of articles and the process has been shown in Figure 1. 

Discussion

KMC in LMICs

A significant fraction of LBW newborns worldwide are delivered in LMICs, including in India [22]. As estimated, over 20 million childbirth of LBWs takes place annually in the whole world, with 18 million of those births occurring in LMICs. Compared to HICs, the prevalence of LBW infant mortality is greater in LMICs [6]. Newborns in HICs have access to a variety of neonatal support services, including specialist medical personnel and technical resources. Once stabilized, this frequently entails neonatal intensive care units (NICUs) [23]. Each year, more than 50% of the LBWIs that are born in LMICs do not survive compared to their counterparts born in high-income countries [6,24]. The high death rate of LBW babies in LMICs is associated with poverty, poor health/illness, unhygienic behavior, and lack of proper healthcare facilities [4]. In comparison to HICs, many LBW infants in LMICs, particularly those living in rural areas, lack access to NICUs, which are facilities with specialized medical personnel and equipment for stabilizing physiological processes including temperature, breathing, and other vital signs in neonates [23]. Even now a sizable majority of childbirths take place at home, and there is limited access to medical facilities [22]. KMC has been demonstrated to be a low-cost affordable method, a human approach to caring for and surviving the LBW neonates in LMICs [25]. KMC might be an effective way for hospitals to employ their limited resources in LMICs [26].

Benefits of KMC to LBW babies/infants

Practicing KMC may lead to an increase in the survival of LBW newborns. Evidence suggests that KMC lowers mortality, probably by promoting better thermoregulation, enabling earlier breastfeeding, lowering the risk of nosocomial infection i.e. hospital-acquired infection, lowering the risk of obstructive sleep apnea, and enhancing mother-infant bonding [27]. The application of KMC in LBW infants might reduce the length of hospital stay.

The WHO advocates KMC application to LBW babies, including those that need medical care such as IV fluids, medications, or oxygen, and it is seen to improve conditions in such babies [28]. KMC promotes the growth and development of LBW babies [20]. Early KMC provides tactile stimulation to babies. Breastfeeding and nipple sucking also activate their sensory systems, which are afferent, kinetic, and olfactory. Clinical health professionals also anticipate that the adoption of KMC would strengthen the emotional ties between mothers and their newborns. It enhances mother-child connection, bonding, and attachment, all of which are crucial for the child's social and emotional growth [29]. Newborns feel safer as a result, and mothers and their babies were more at ease.

Benefits of KMC to Mothers

The mothers of LBW babies mostly show symptoms of stress, anxiety, and depression. Most of the studies have demonstrated that KMC has a favorable effect on maternal mental health [30]. Psychological symptoms such as postpartum depression and other depressive factors like suicidal tendencies or suicidal thoughts can be prevented in mothers by performing KMC. Physical symptoms seen in mothers of LBW infants including tiredness and fatigue get reduced by performing KMC [23]. Other symptoms like anxiety and sleeping disorders like insomnia in mothers can be reduced by practicing KMC. Mothers might also feel more confident in their abilities. KMC can be a helpful intervention for the mother, as well as for the LBW babies [31].

Difficulties Faced by Mothers While Performing KMC

Postpartum depression affects roughly one n five women in LMICs, and the risk is higher for mothers of LBW infants [32]. When taking a look at all papers and publications from LMICs, "pain/weariness" is a major obstacle in performing KMC [31]. This included the inability to hold the infant because it was too heavy or uncomfortable, pain in the chest or back, and weariness. Other difficulties included positioning concerns such as having trouble sleeping with the baby on your chest, temperature discomfort, and problems with clothing and medical devices. Mothers' health problems also provide a significant obstacle to the practice of KMC. This may include general maternal diseases, postpartum depression, episiotomy repair pain, and cesarean section recovery. These obstacles indicate that it may be very difficult for women to practice continuous KMC, especially those who have less motivation and have medical/health issues [31].

Positioning of the Baby and the Mother/Caregiver during KMC

As per the WHO, in KMC the mother exclusively breastfeeds the child while holding him or her in skin-to-skin contact on her chest between her bare breasts until the child no longer prefers to be in that position [33]. Neonates ought to be positioned between the mother's breasts with the head moved to the side to allow for breathing and arms a bit extended (sniffing position). This little bit of stretched head position maintains the airway open and permits the mother and child to make eye contact (initiate let-down reflex) [8]. The arms should also be flexed, and the hips should be bent and abducted in a "frog"-like position. The infant’s abdomen ought to be at the level of the mother's epigastrium (diapers should not be put on because they could prevent skin-to-skin contact) [8]. The mother/caregiver can provide KMC to LBW infants in upright, prone, and inclined positions. Sometimes they can provide KMC while seating on a chair or walking around also [34].

KMC Trainers in LMICs

Regardless of the lack of widespread knowledge about KMC, research from LMICs reveals that it is simple to educate mothers about the KMC procedure [31]. For instance, an Indian training program discovered that 88% of mothers could comprehend KMC after just one training session [35]. In hospitals, healthcare providers such as nurses, especially pediatric nurses, guide the mothers about the importance of KMC and also help them in providing KMC in the neonatal and maternal wards or ICUs. Tutoring of service delivery workers at all pertinent levels is necessary for the launch and the growth of KMC [13]. KMC trainers must have experience in taking care of LBW newborns and a background in maternal and neonatal health care and services [13]. The study team regarding KMC includes anthropologists and social scientists while a KMC trainer team includes doctors, and health workers such as Auxiliary Nurse Midwives (ANMs), Accredited Social Health Activists (ASHAs) as well as traditional birth attendants (TBAs)/dais, the workers who assist mothers and families in home deliveries) [22,13].

 *Involvement of Father in KMC in LMICs*

Some studies show that fathers also provide KMC in LMICs. If the mothers are not able to provide KMC to LBW infants due to some circumstances then fathers can provide it. In fact, infants who received KMC from fathers had better state behavior responses and maintained greater skin temperatures [36]. Fathers who administered KMC were more eager to participate in baby care, increasing bonding and attachment with the child, and creating a more energizing, peaceful, and all-around healthier family atmosphere, which is good for the development of LBW infants [36]. Qualitative research revealed that fathers love the feeling of being needed and getting involved in the process of KMC, which helped them to perform a paternal role and also get equality in performing parenthood [36].

Family Practicing KMC in LMICs

In LMICs, family, friends, relatives, neighbors, and other parents are known as the best promoter of practicing KMC [31]. KMC practice involves both the mother's drive and the perception that KMC-based infant care at home is simple. The need for expensive LBW care can be reduced or even eliminated by family readiness in implementing KMC, which can be done by families. Creating an emotional connection with the baby is another benefit of the KMC approach for parents and other caregivers [22]. Before leaving for home from the hospital, KMC practice should be discussed with a counselor. A health education activity for parents and the baby's family is making the families ready to use KMC in the house. This can boost family confidence in caring for LBW babies [9].

*KMC in India* 

India is a lower-middle-income country. KMC was established in India in 1994, and as part of the Newborn Action Plan, it became a nationwide guideline in 2014 [37]. According to studies, there are several obstacles in the execution process in India, including difficulty in identifying LBW infants for KMC, insufficient space, and equipment in institutions for KMC service, lack of medical staff, and a lack of understanding and expertise to perform and assist in KMC. Various studies show that mothers respond favorably to KMC, and families embrace giving KMC at home [37]. Table 1 summarizes the results of some research articles related to KMC.

**Table 1:** Summary of Articles Related to Kangaroo Mother Care

Authors	Year	Results of some Research Articles Related to KMC
Murty AI [10]	2018	According to this study, it is seen that there is a successful implementation of KMC in families at the community level.
Moore et al. [12]	2016	The use of KMC to encourage breastfeeding. Establishing potential dose-response effects, optimal initiation timing, and physiological benefits for newborns throughout the transition to extra-uterine life is the main goal of this study.
Conde-Agudelo [14]	2014	This updated review encourages using KMC in place of traditional newborn care for LBW infants, especially in places with limited resources. More research is necessary for the long-term neurocognitive outcomes and the safety and effectiveness of early onset continuous KMC in LBW newborns.
Mekonnen et al [15]	2019	KMC encourages prior initiation of breastfeeding. As a result, hospitals must implement the KMC program for premature and LBW babies.
El-Farrash et al. [18]	2020	Preterm infants who get KC for extended periods reach full enteral feeds more quickly, breastfeeding gets successful, perform better neurologically and behaviorally, have better temperature regulation, and have better tissue oxygenation.
Jayaraman et al. [19]	2017	According to this study, Early KMC dramatically boosted direct breastfeeding and exclusive human milk feeding in LBW newborns.
Winkler et al. [24]	2020	This study shows the benefit of KMC in rural areas with limited resources. Results are better than those reported for comparable groups not performing KMC in rural sub-Saharan Africa and are equivalent to KMC programs in metropolitan regions when neonates start KMC after stabilization.
Bajaj et al [25]	2015	By focusing appropriately on the areas where knowledge, attitude, and practices are deficient, the results of KMC can be improved. The level of knowledge in this study seemed to not affect on either attitude or practice.
WHO Immediate KMC Study Group [27]	2020	The results of this trial will have global repercussions in the future, not only on the postpartum treatment of LBW newborns but also on the adoption of the mother-neonatal intensive care unit (M-NICU) paradigm for NICU architecture.
Landry et al. [30]	2022	Mothers who engaged in mindfulness exercises while providing kangaroo care (KC) reported that Mindful Kangaroo Care (MKC) was acceptable, and practical, and reduced their stress, anxiety, and depression.
Seidman et al. [31]	2015	This systematic review's goal was to discover practicing enablers as well as the most commonly mentioned obstacles to KMC adoption.
Mazumder et al/ [33]	2017	Neonatal mortality post-enrolment and mortality occurring between enrolment and 6 months of age are the primary outcomes of this study. The secondary outcomes include breastfeeding practices, the prevalence of diseases and seeking medical attention for the same, hospitalizations, increase in weight and length, and, in a subsample, neurodevelopment.
Deng et al. [36]	2018	The study’s purpose is to know the security and consequences of paternal SSC on preterm infants' neurocognitive outcomes.
Rasaily et al [38]	2017	According to the study, it was possible to offer KMC using the current infrastructure, and the majority of mothers of LBW infants considered the method to be acceptable.

Recommendations

Need to Promote KMC in LMICs

In LMICs, there is a greater ratio of mortality and morbidity rate of LBW/premature babies. Due to the lack of proper medical access in hospitals, newborn deaths occur. For the survival of LBW babies, implementation of KMC is a must.

Measures to Promote KMC in LMICs

The entire hospital staff must be on board with the concept and take part in creating the KMC policy and procedure for the adoption of KMC to be successful. Every mother ought to be aware of KMC. It is essential that the general population is aware of KMC and understands its advantages [39].

The media plays an essential role in promoting KMC. Promotion of KMC in LMICs is done by spreading knowledge about KMC in schools, featuring KMC in the media, particularly on TV and in the neighborhood newspaper, bringing up KMC's advantages in the press, particularly on the radio and in periodicals, and displaying KMC materials in primary care clinics through posters or videos [39].

Governmental Strategies for KMC in India

The Government of India is committed to improving child health by prioritizing newborn care services that increase child survival. In 2014, the Child Health Division of the Ministry of Health and Family Welfare (MOHFW) released the National Guidelines for Kangaroo Mother Care (KMC) and Optimal Feeding of Low Birth Weight Infants in an effort to implement KMC at the facility level. The MOHFW allotted funds to each state for the adaptation of KMC spaces within the special newborn care units (SNCUs) [40]. The IXth International KMC Conference was held in Ahmedabad in 2012 [41]. A KMC Foundation was established in India, and UNICEF and the Indian government are paying more attention to promoting KMC in neonatal facilities [41]. The Indian government promotes KMC through programs such as Navjaat Shishu Suraksha Karyakram (NSSK) and Facility-Based Integrated Management of Newborn and Childhood Illnesses (F-IMNCI) [42]. To attain single-digit neonatal mortality by 2030, KMC works as a priority mediator in the "India Newborn Action Plan", a strategy document of the Indian government. Operating guidelines for facility-based KMC from the Ministry of Health and Family Welfare, Government of India, emphasize the value of maintaining KMC at home in conjunction with home-based care and having an ASHA worker check in on the infant [42].

Policy Formulations and Guidelines

As per the WHO publication, *Kangaroo Mother Care: A Practical Guide*, a national policy guarantees a cogent and efficient integration of the profession within the already-existing health system, education, and training frameworks [28]. National standards and norms for the care of LBW babies must be developed. Clear monitoring and evaluation criteria must be included in the standards. The ideal way to create these is with the assistance of parents and pertinent professional groups [28]. The KMC policy is a written declaration that outlines the advantages of KMC and commits the service to put it into practice and promote it. It doesn't have to be a drawn-out, difficult document [34]. Each health facility implementing KMC should have a documented policy and set of guidelines customized to the local circumstances and cultural norms. When possible, the entire team should be involved in drafting local procedures based on national or international recommendations. This will increase the effectiveness of the policies and guidelines [28]. Obtaining copies of the KMC policy and rules from another service where KMC is successfully implemented may be helpful. KMC should be marketed as a cost-efficient, reliable, and safe way to care for newborn babies [40]. Monthly staff meetings will be beneficial after the KMC protocol is implemented to discuss and analyze data and problems and, if required, enhance the protocol [28].

## Conclusions

 In LMICs, there is a greater prevalence of LBWs than in the HICs. Preterm birth is the main cause of low birth weight. KMC is the procedure carried out on LBW infants for their survival. Both parents and other family members can provide KMC to the LBW baby after the stabilization of the baby. KMC is very beneficial for LBW infants, mothers, fathers, and families. It reduces the mortality and morbidity rate of LBW infants as well as promotes the growth of the newborn. Hospital stays are also get decreased by the application of KMC. Providing KMC can build an emotional bond between the LBW infants and their parents. It is a cost-effective method that doesn’t need any kind of equipment. Community health workers such as ASHAs provide training and health education related to KMC. It increases the confidence of parents in caring for LBW babies.
